# Publisher Correction: IAPP toxicity activates HIF1α/PFKFB3 signaling delaying β-cell loss at the expense of β-cell function

**DOI:** 10.1038/s41467-019-11516-y

**Published:** 2019-07-31

**Authors:** Chiara Montemurro, Hiroshi Nomoto, Lina Pei, Vishal S. Parekh, Kenny E. Vongbunyong, Suryakiran Vadrevu, Tatyana Gurlo, Alexandra E. Butler, Rohan Subramaniam, Eleni Ritou, Orian S. Shirihai, Leslie S. Satin, Peter C. Butler, Slavica Tudzarova

**Affiliations:** 1 Larry L. Hillblom Islet Research Center, David Geffen School of Medicine, University of California, Los Angeles, Los Angeles, CA 90024 USA; 2 Department of Pharmacology and Brehm Diabetes Research Center, University of Michigan, Ann Arbor, MI 48105 USA; 3 Division of Endocrinology, Department of Medicine, David Geffen School of Medicine, University of California, Los Angeles, Los Angeles, CA 90024 USA; 4 Jonsson Comprehensive Cancer Center, David Geffen School of Medicine, University of California, Los Angeles, Los Angeles, CA 90024 USA

**Keywords:** Mechanisms of disease, Type 2 diabetes

Correction to: *Nature Communications* 10.1038/s41467-019-10444-1, published online 08 June 2019.

The original version of this Article omitted the following from the Acknowledgements:

Dr. Butler’s grant was funded by the National Institute of Diabetes and Digestive and Kidney Diseases, #DK059579.

This has now been corrected in both the PDF and HTML versions of the Article.

