# Supplementary material for: IAPP toxicity activates HIF1α/PFKFB3 signaling delaying β-cell loss at the expense of β-cell function
Source: Nat Commun. 2019 Jun 18;10:2679. doi: 10.1038/s41467-019-10444-1 (PMC6581914; doi:10.1038/s41467-019-10444-1)
Supplement: Supplementary file 1 — Supplementary Information [file 41467_2019_10444_MOESM1_ESM.pdf]

IAPP toxicity activates HIF1α/PFKFB3 signaling delaying β-cell loss at the expense of β-cell function

Montemurro et al.

Supplementary Figure 1

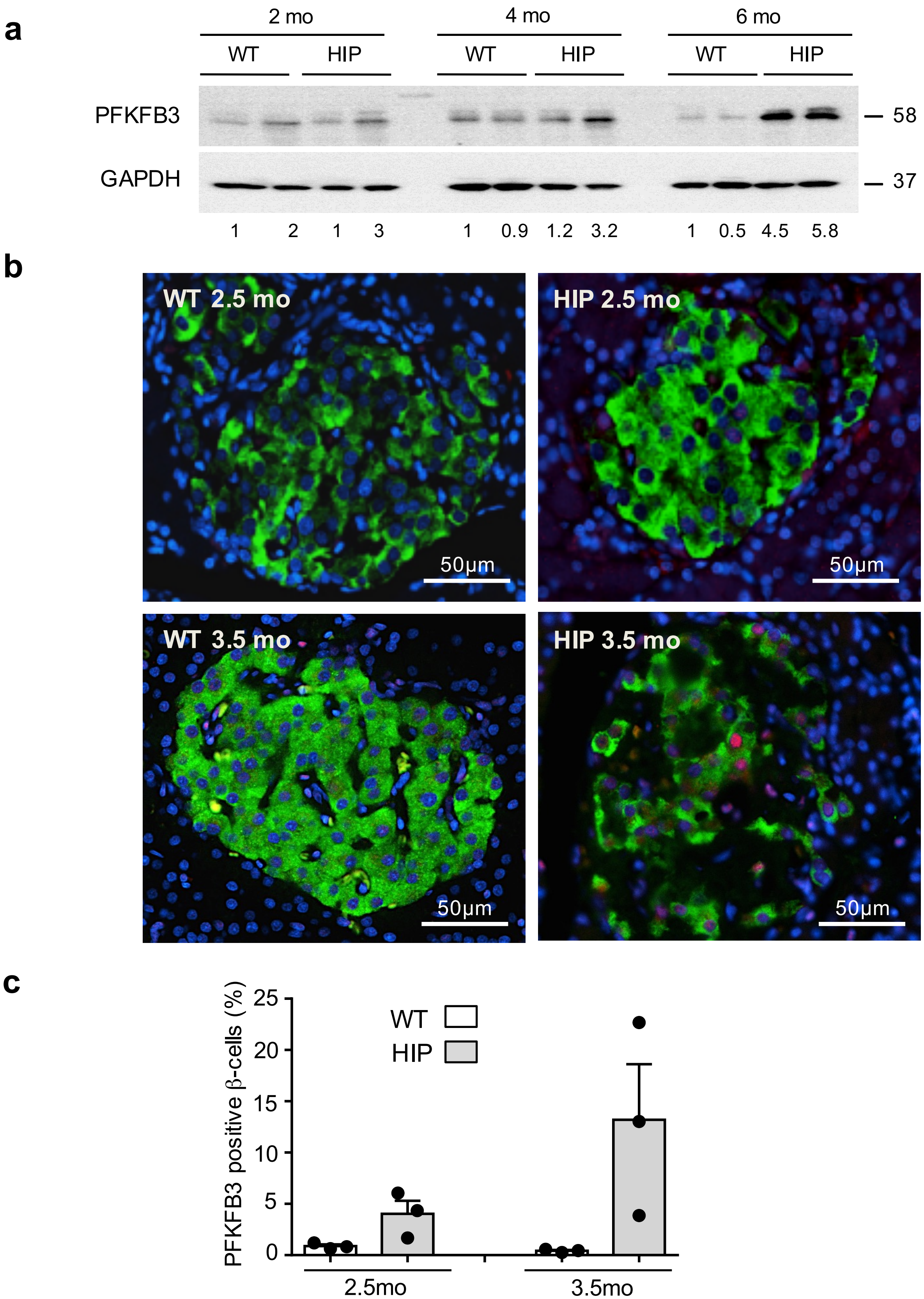

Supplementary Figure 1. PFKFB3 is upregulated in prediabetic HIP rats of 3.5 months of age. (**a**) Representative Western blot of PFKFB3 in whole cell extracts from WT and HIP islets. (**b**) Representative immunofluorescence images of islets from WT and HIP rats at 2.5 (upper panel) and 3.5 (lower panel) months of age stained for PFKFB3 (red), insulin (green) and nuclei (blue). (**c**) Frequency of PFKFB3 positive β-cells in HIP vs. WT rats (2.5 and 3.5 months). Data are presented as mean ± SEM, n=3 for each group.

Supplementary Figure 2

a

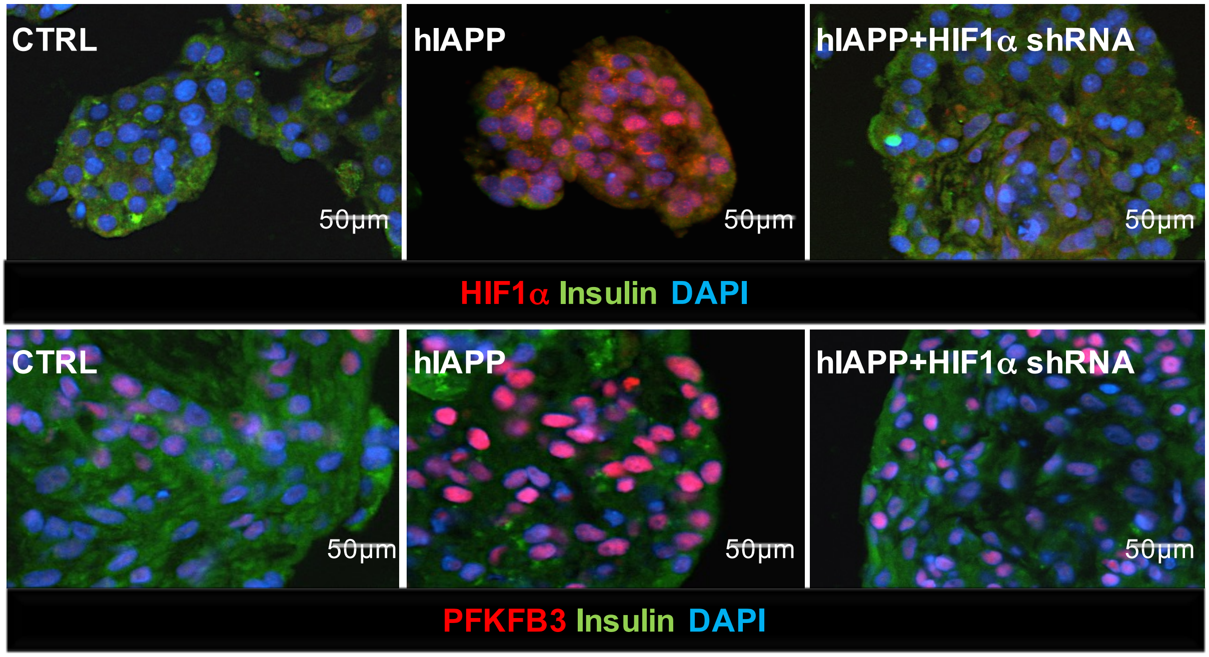

b

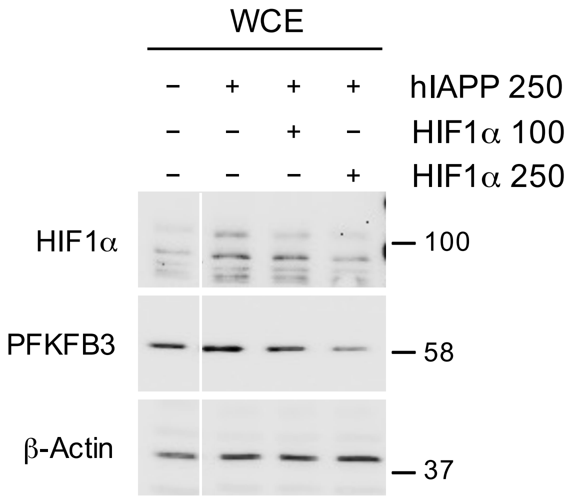

Supplementary Figure 2. Silencing of HIF1α in human islets suppresses PFKFB3 expression levels. (**a**) HIF1α (upper panel) and PFKFB3 (lower panel) immunostaining of human non-diabetic islets transduced with LacZ-AdV (CTRL) or hIAPP-AdV for 48h with or without HIF1α shRNA. HIF1α or PFKFB3 is in red, insulin in green and nuclei in blue. (**b**) Immunoblotting analysis of whole cell extracts prepared from human non-diabetic islets transduced with hIAPP-AdV for 48h with or without HIF1α shRNA (100 or 250 MOI).

Supplementary Figure 3

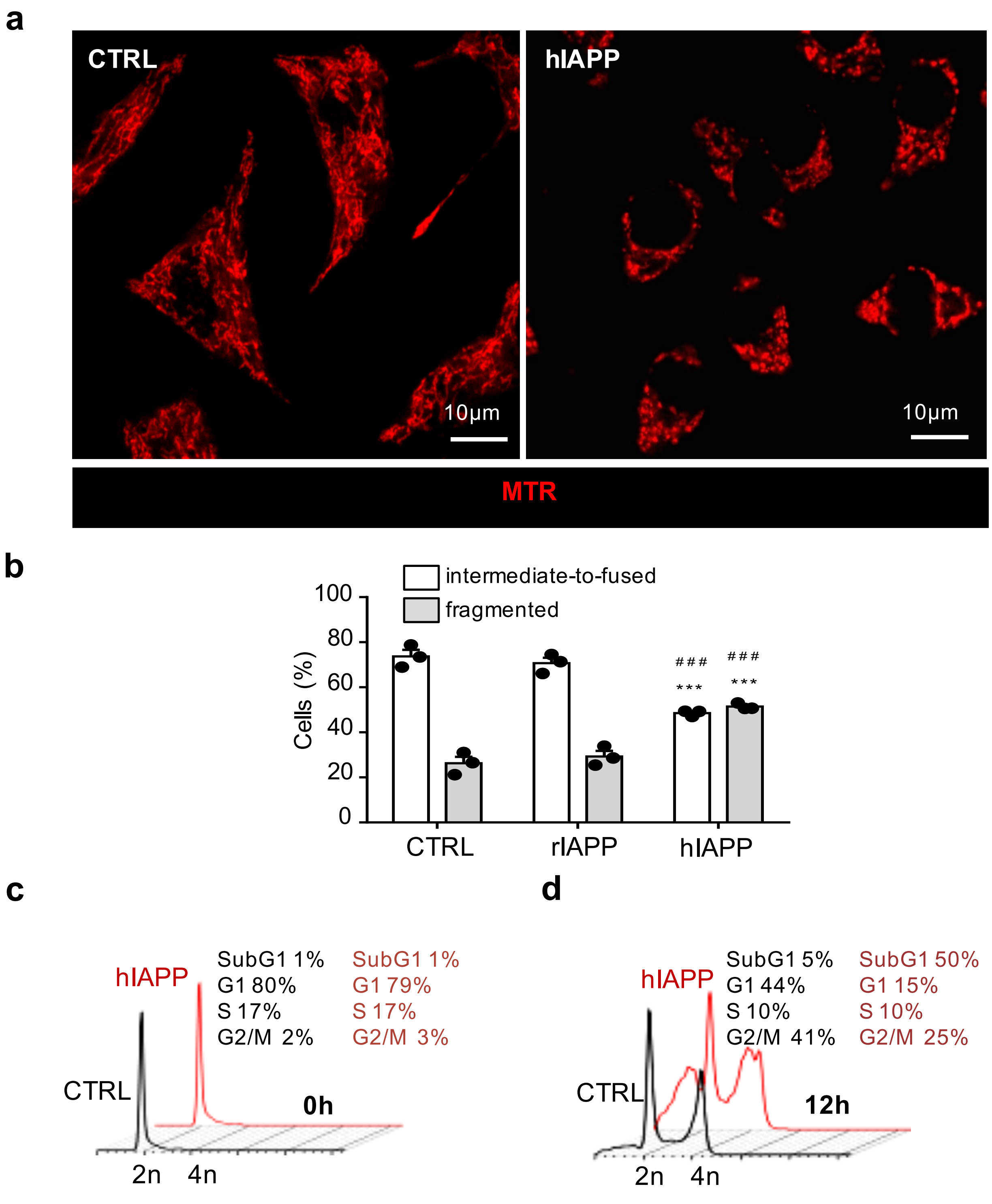

Supplementary Figure 3. hIAPP induces fragmentation that precedes cell death in INS 832/13 cells synchronized at G1/S. (**a**) Representative images of INS 832/13 stained with Mitotracker Red (mitochondria, red) and DAPI (nuclei, blue). Cells were cultured in RPMI medium, synchronized at G1/S of the cell cycle and transduced for 36h with adenoviral vectors expressing LacZ (CTRL) or hIAPP. (**b**) Quantification of fragmented (>50%) and intermediate-to-fused (>50%) mitochondria presented as a percentage of all mitochondria. FlowJo overlay of flow cytometry diagrams from INS 832/13 cells at (**c**) 0h and (**d**) 12h post-release from aphidicolin block showing subG1 accumulation at 12h (36h treatment with hIAPP-AdV). Data are presented as mean ± SEM, n=3 for each group. Statistical significance in b was analyzed by one-way ANOVA test with Tukey's post-test (***p<0.001 vs CTRL, ###p<0.001 vs rIAPP).

Supplementary Figure 4

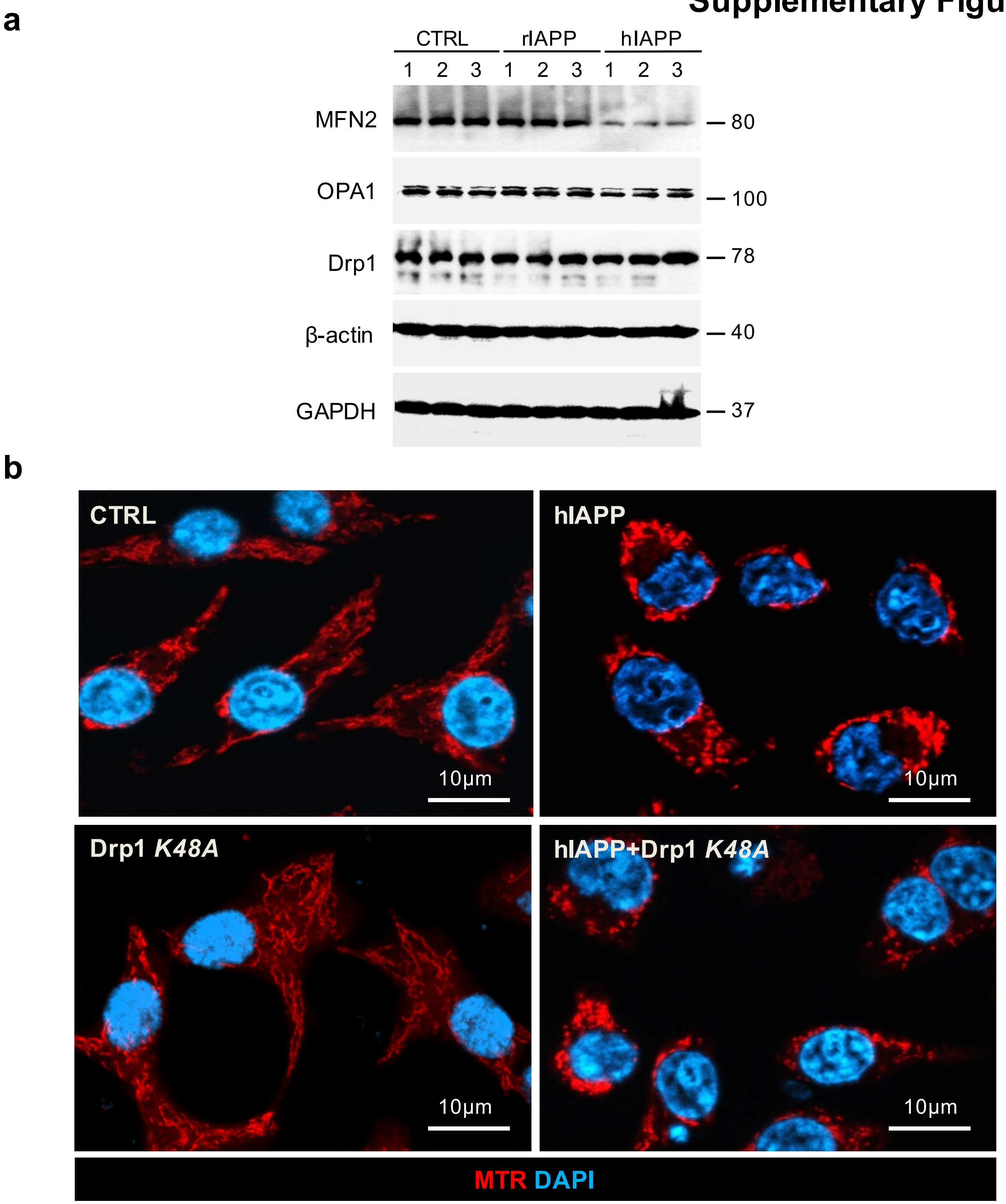

Supplementary Figure 4. hIAPP affects mitochondrial fusion by reducing MFN-2 levels but not mitochondrial fission. (**a**) Immunoblotting of indicated dynamin-related proteins in untreated (UT), control (CTRL, LacZ) and hIAPP overexpressing INS 832/13 cells. (**b**) Representative images of CTRL-LacZ and hIAPP-AdV transduced INS 823/13 β-cells in presence or absence of the dominant negative Drp1*K48A* mutant . Mitochondria were stained with mitotracker red (red) and nuclei with DAPI (blue).

Supplementary Figure 5

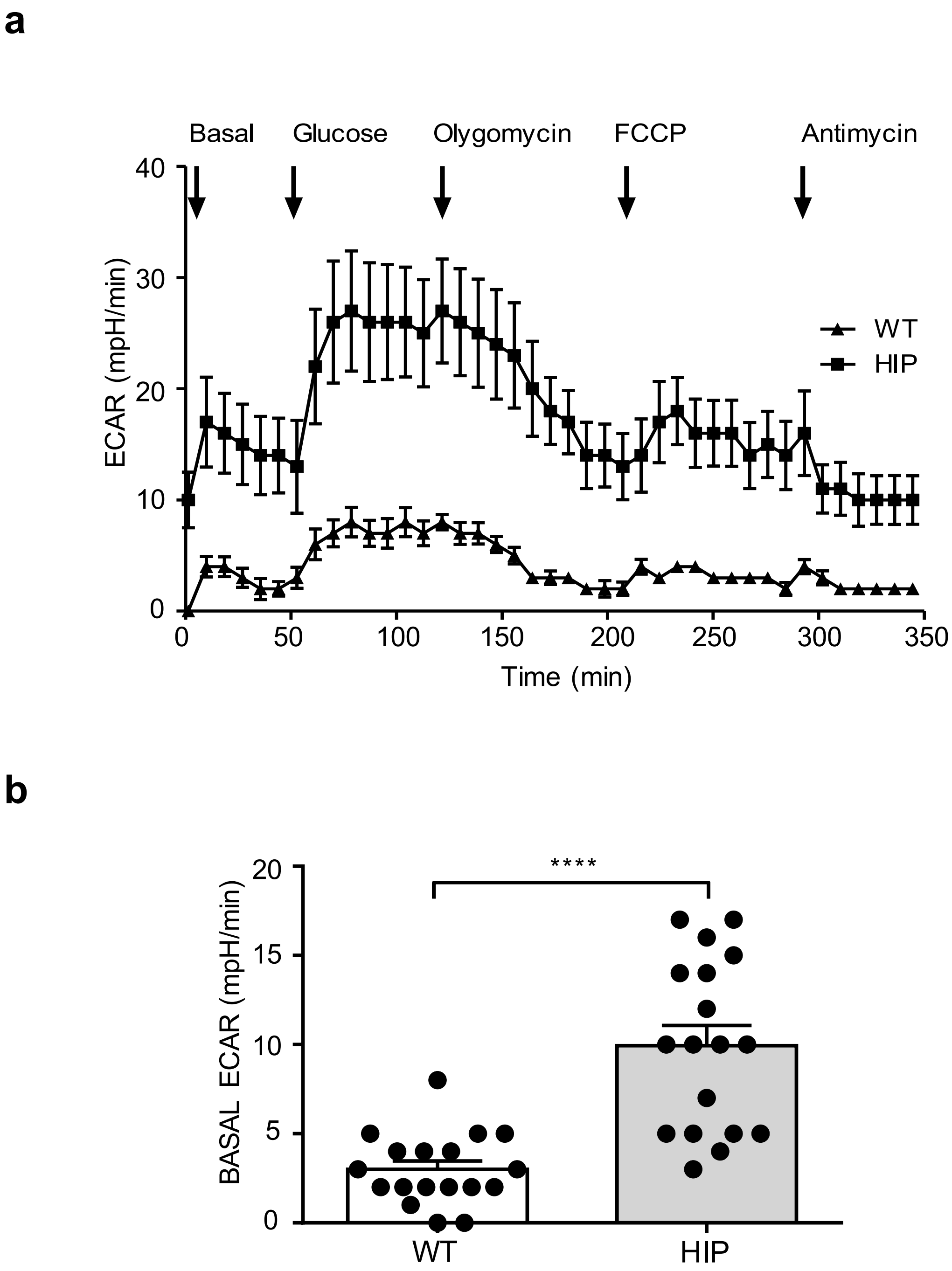

Supplementary Figure 5. Effect of hIAPP on extracellular acidification rate in rat islets. (**a**) Profiling of extracellular acidification rate (ECAR, mpH/min) in islets from WT and HIP rats measured with the Seahorse Bioscience XF24 extracellular flux analyzer. n=3 for each group. (**b**) Quantification of basal ECAR in islets of comparable surface area from WT and HIP rats. n=18 for each group. Data are presented as mean ± SEM. Statistical significance in b was analyzed by Student t-test (****p<0.001)

Supplementary Figure 6

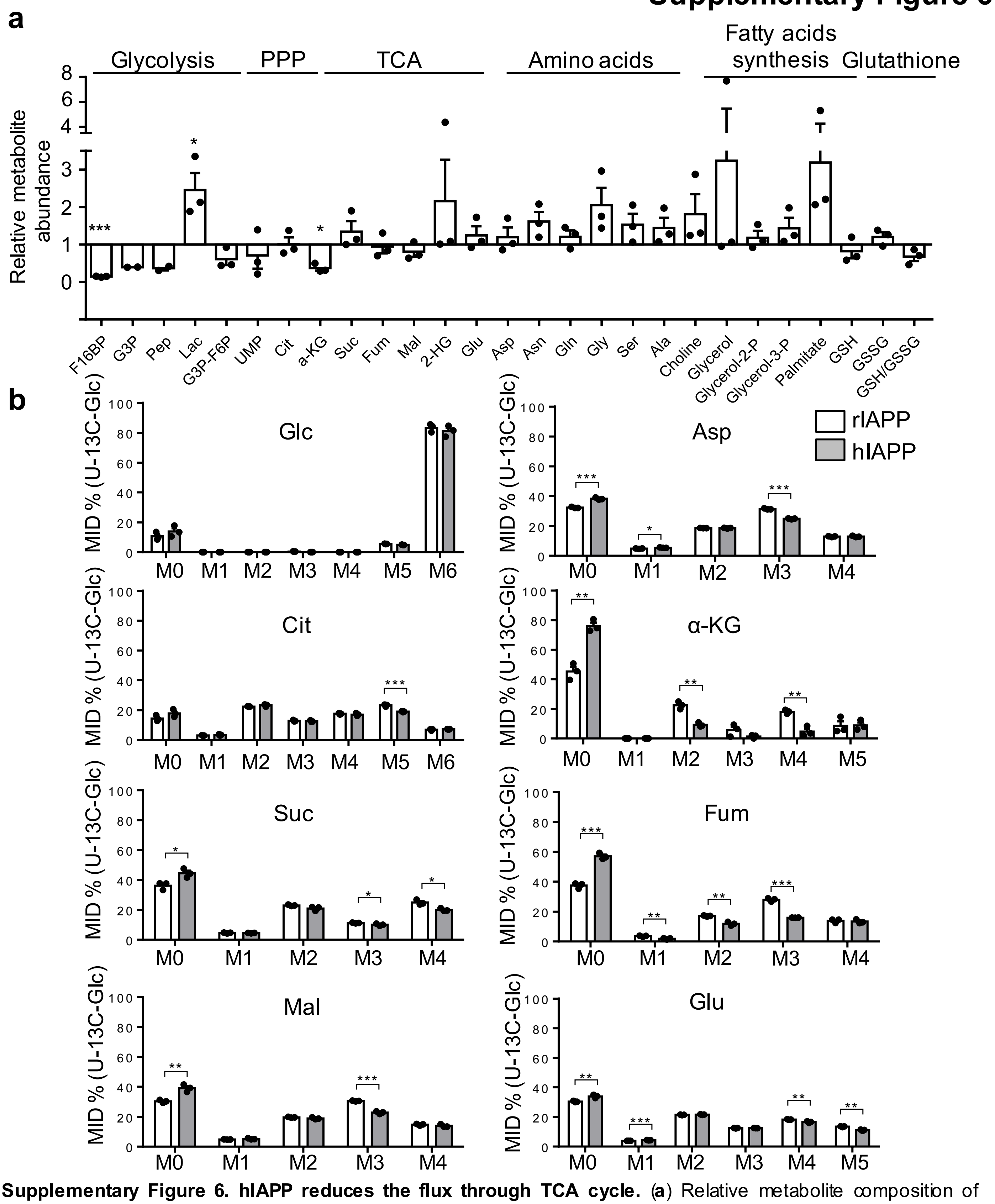

Supplementary Figure 6. hIAPP reduces the flux through TCA cycle. (**a**) Relative metabolite composition of hIAPP (30h) overexpressing INS 832/13 cells presented as fold change of control rIAPP overexpressing cells. (**b**) Mass isotopomer distribution (MID) of the TCA intermediates derived from culturing INS 832/13 with [U-$^{13}C_6$] glucose; Glucose – Glc; Aspartate – Asp; Citrate – Cit; α-ketoglutarate – α-KG; Succinate – Suc; Fumarate – Fum; Malate – Mal; Glutamate – Glu. Data are presented as mean ± SEM, n=3 for each group. Statistical significance in b was analyzed by Student t-test (*p<0.05, **p<0.01, ***p<0.001).

Supplementary Figure 7

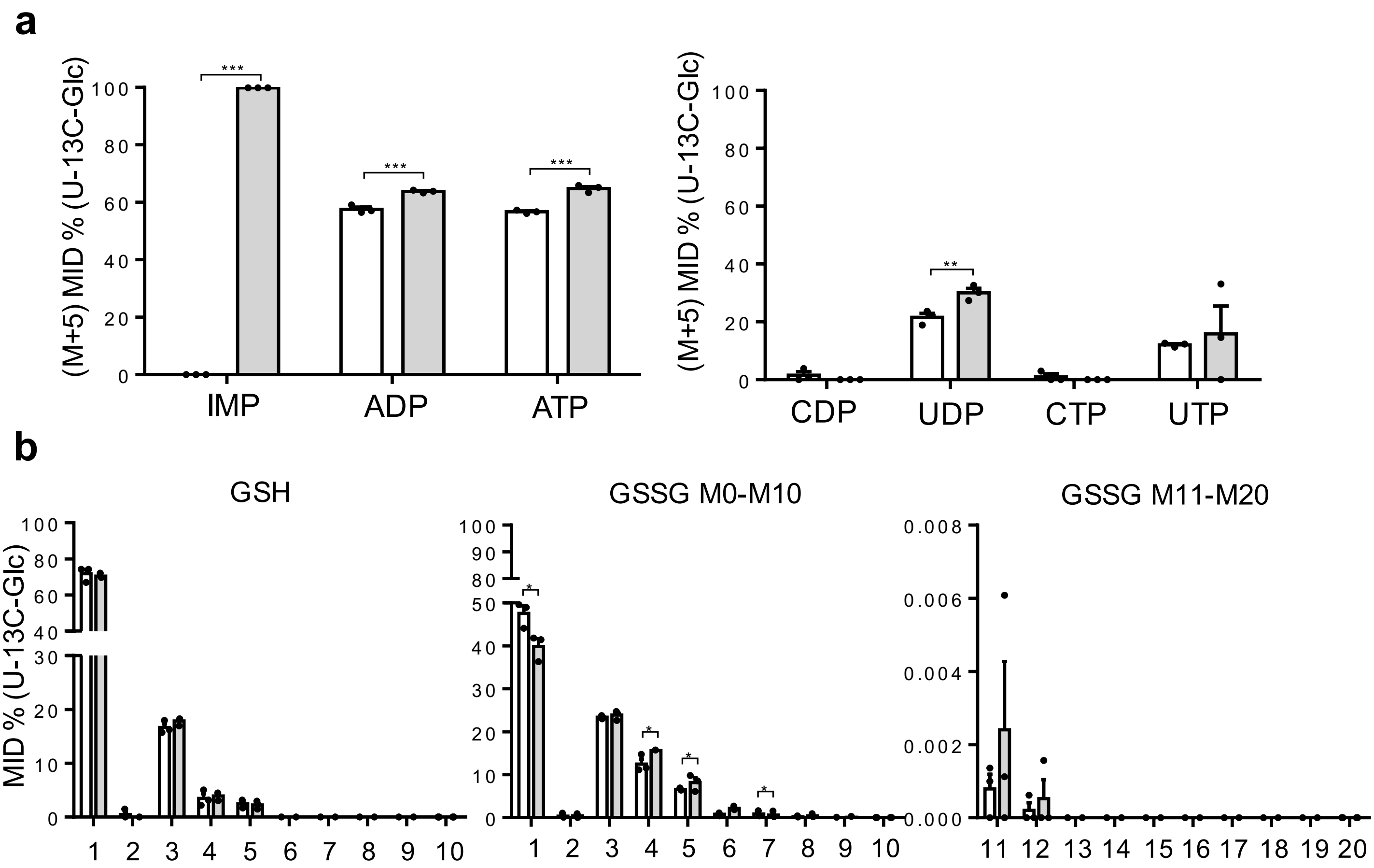

Supplementary Figure 7. hIAPP increases de novo purine synthesis and oxidized glutathione. (**a**) Mass isotopomer distribution (MID) of the TCA intermediates derived from culturing INS 832/13 with [U-$^{13}C_6$] glucose. Inositol monophosphat – IMP; Adenosin diphosphate – ADP; Adenosin triphosphate – ATP; Cytidin diphosphate – CDP; Cytidin triphosphate – CTP; Uridin diphosphate – UDP; Uridin triphosphate – UTP (**b**) reduced Glutathione – GSH; oxidized Glutathione – GSSG. Data are presented as mean ± SEM, n=3 for each group. Statistical significance was analyzed by Student t-test (*p<0.05, **p<0.01, ***p<0.001).

Supplementary Figure 8

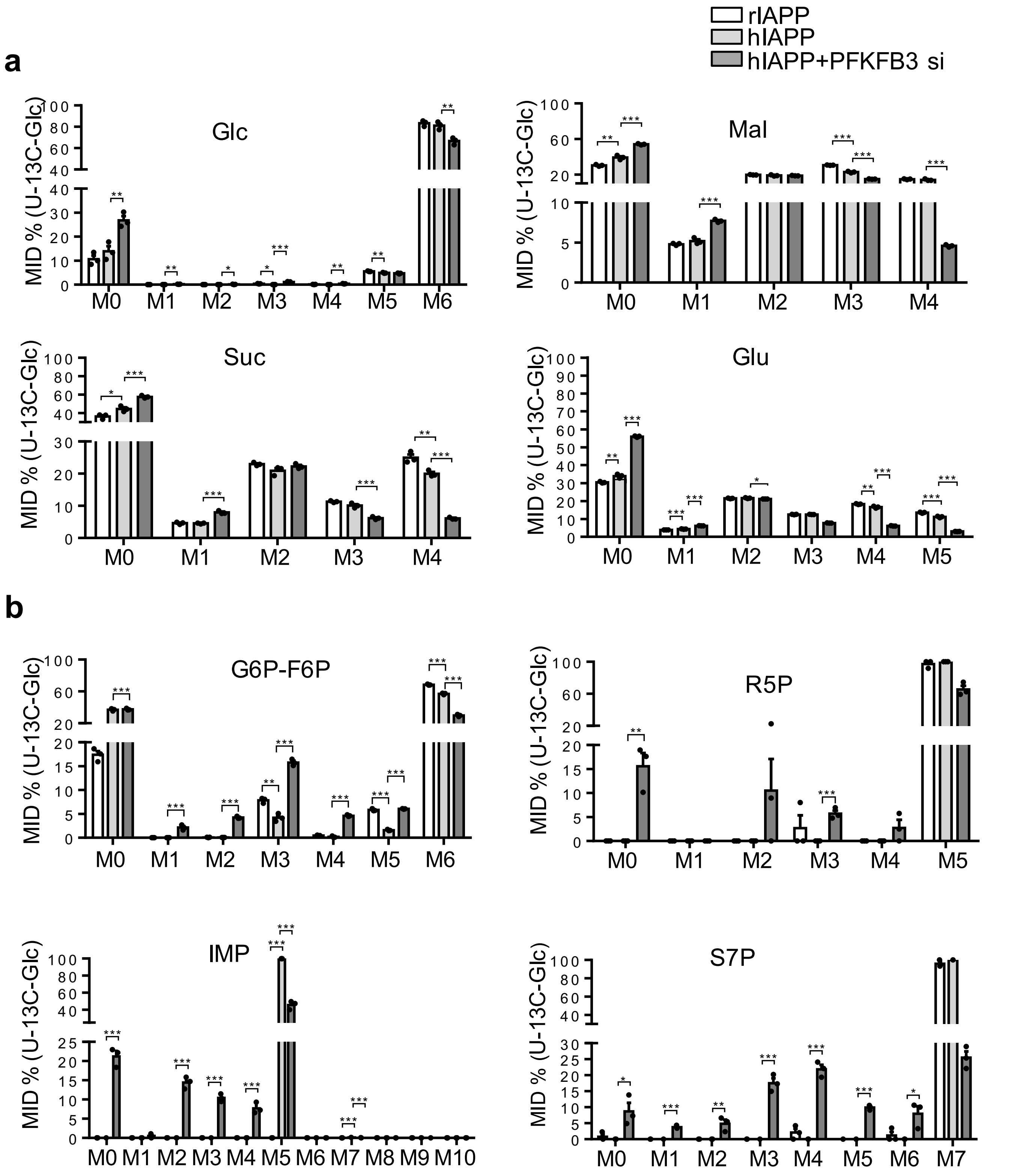

Supplementary Figure 8. Silencing of PFKFB3 stimulates the PPP flux. (**a**) Mass isotopomer distribution (MID) of the TCA intermediates derived from culturing INS 832/13 with [U-$^{13}C_6$] glucose. Glucose – Glc; Malate – Mal; Succinate – Suc; Glutamate – Glu; and the (**b**) pentose phosphate pathway (PPP) metabolites G6P-F6P – Glucose-6-phosphae-Fructose-6-phosphate; R5P-Ribose-5-phosphate; IMP-Inositol monophosphate; S7P-sedoheptulose-7-phosphate. Data are presented as mean ± SEM, n=3. Statistical significance was analyzed by one-way ANOVA test with Tukey's post-test (*p<0.05, **p<0.01, ***p<0.001).

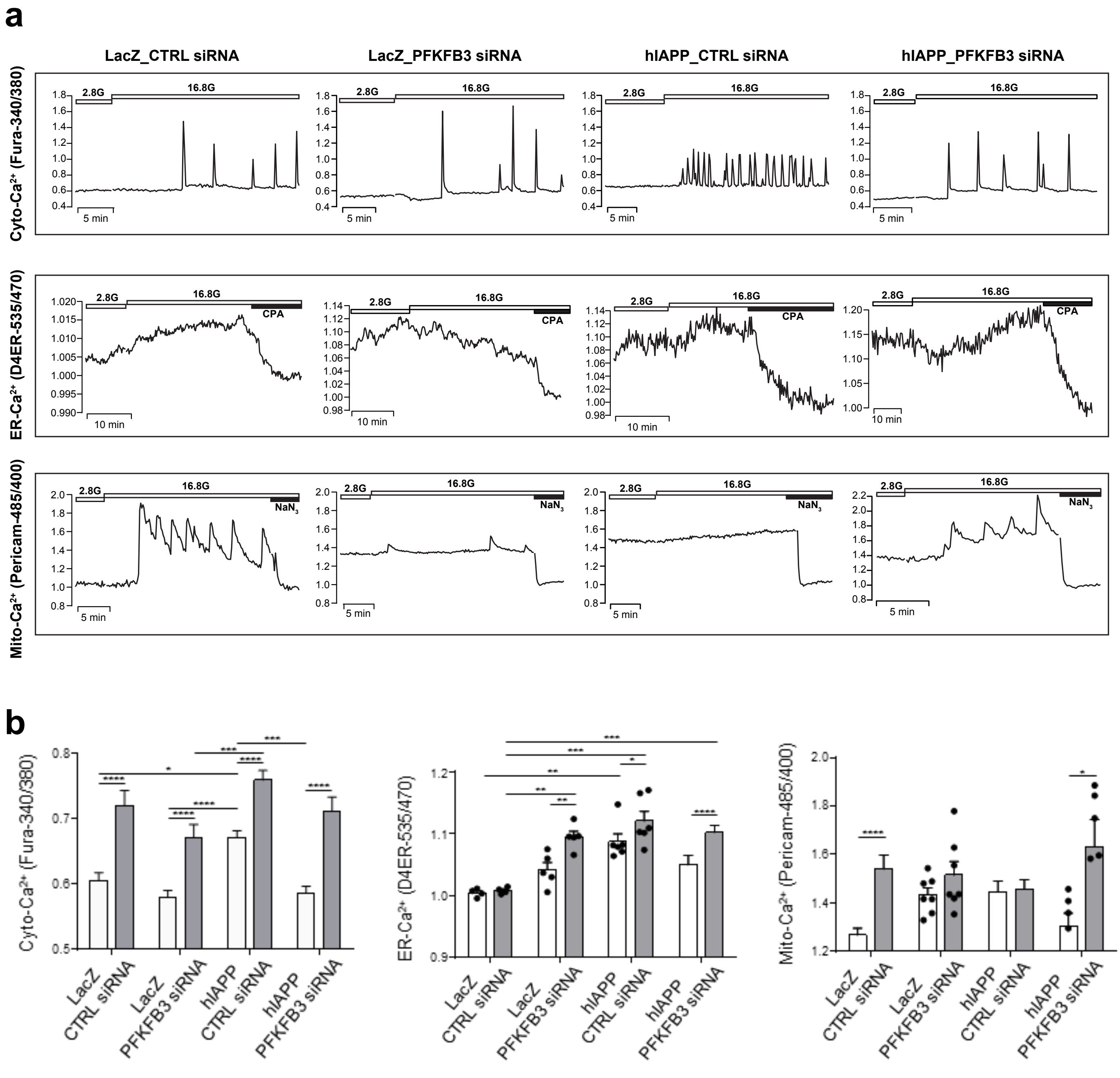

Supplementary Figure 9. Silencing of PFKFB3 recovers Ca2+ levels and organelle dynamics in cells under hIAPP toxicity. (**A**) Representative compartment Ca2+ traces at low (2.8 mM) and high (16.8) glucose as measured in cytosol (FURA-2AM), ER (D4ER) and mitochondria (Mito-Pericam). (**B**) Statistical analysis of compartment Ca2+ using two-way ANOVA. Data are presented as mean ± SEM, n=3. *p<0.05, **p<0.01, ***p<0.005, ****p<0.001.

Supplementary Figure 10

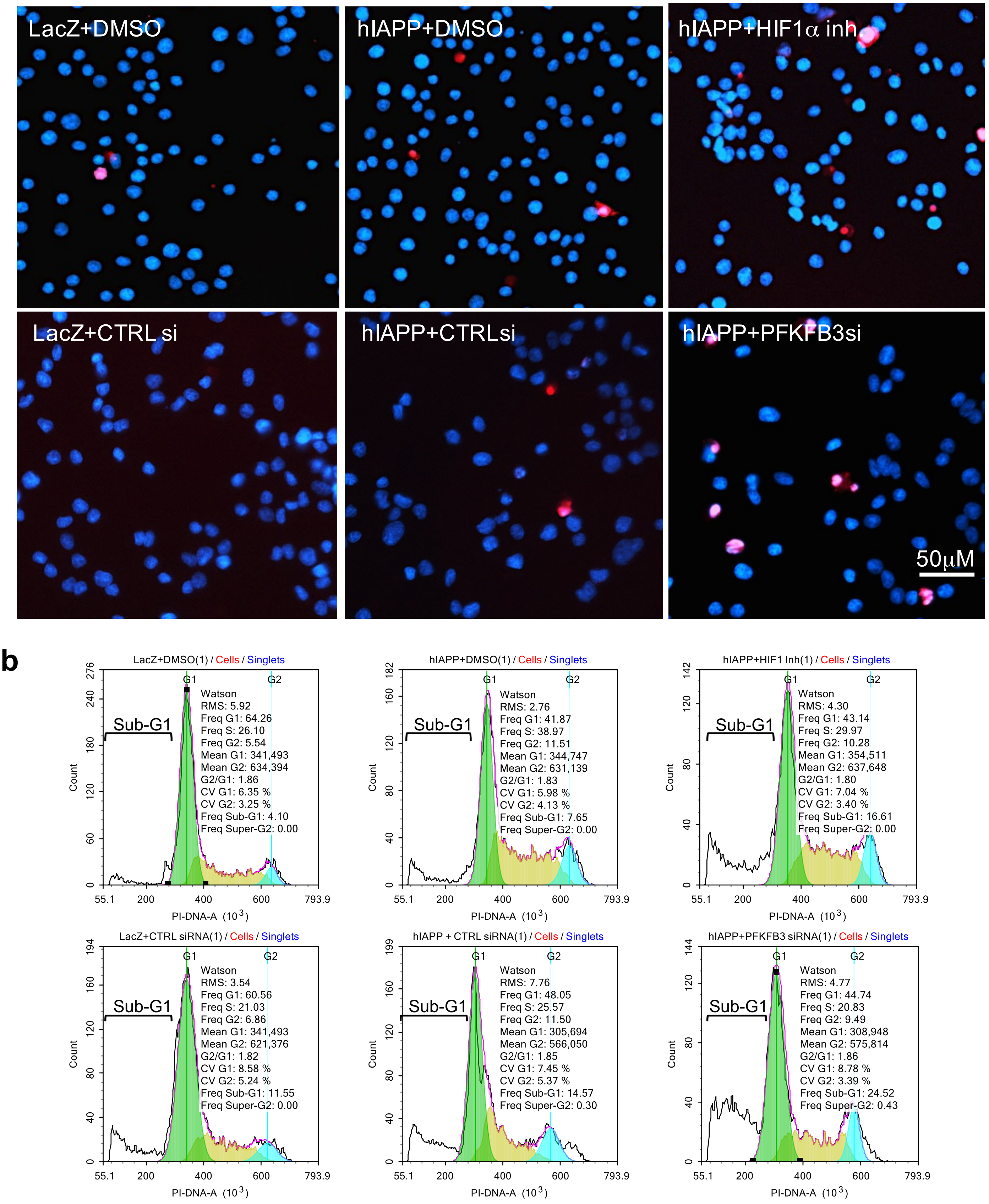

Supplementary Figure 10. Cell death is increased after abrogation of HIF1α/PFKFB3 pathway. (**A**) Representative images of TUNEL staining performed on INS 832/13 treated as described in Fig 9c. TUNEL (cell death, red) and DAPI (nuclei, blue). (**B**) Representative DNA content distribution histograms from FACS analysis of treatments as described under Fig. 9b.

Supplementary Figure 11

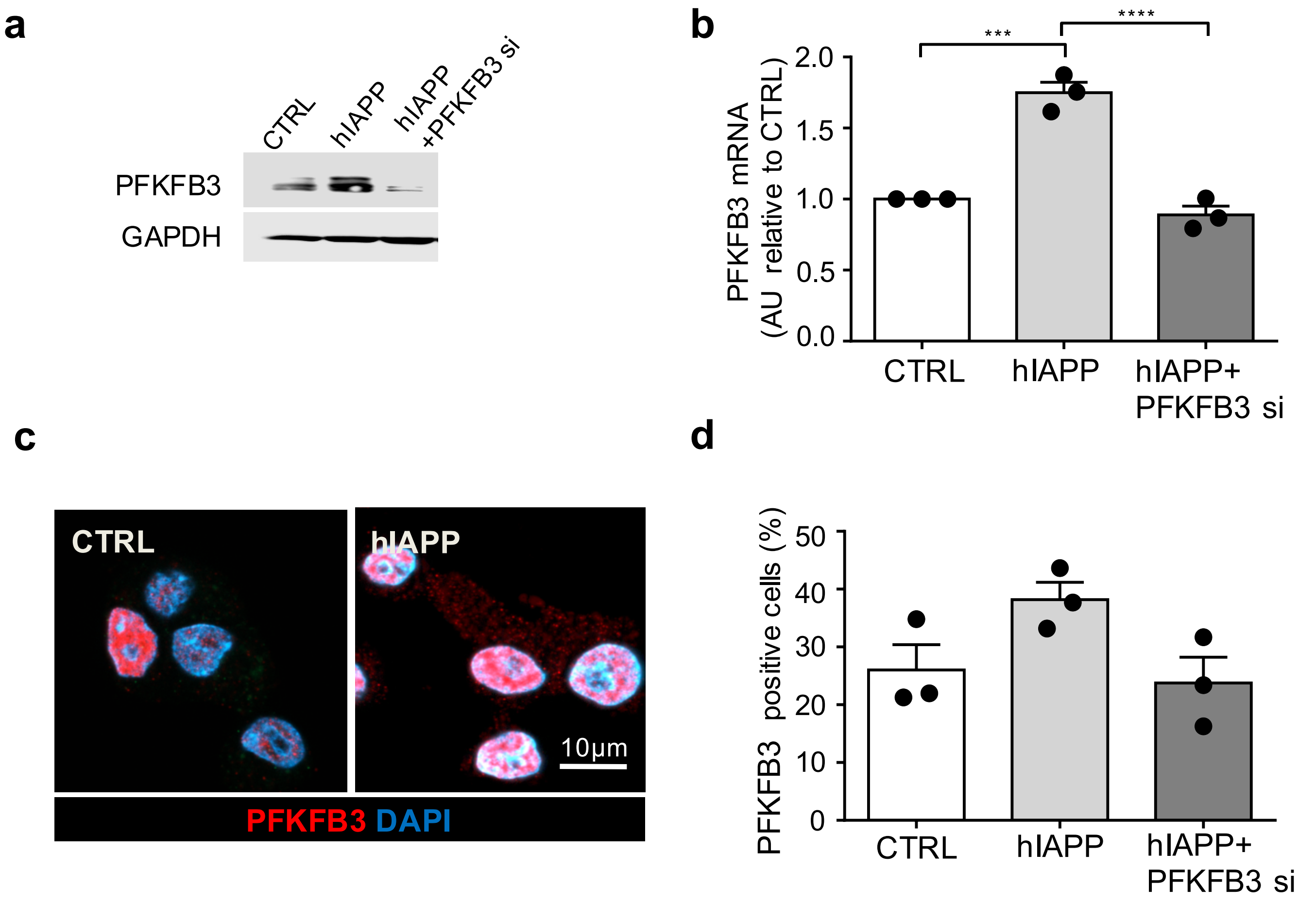

Supplementary Figure 11. Validation of PFKFB3 antibody. (**a**) PFKFB3 protein levels in serum starved INS 832/13 after transduction with LacZ (CTRL) or hIAPP-AdV for 36h with or without PFKFB3 siRNA as assessed by western blot of whole cell extracts. (**b**) Relative PFKFB3 mRNA levels in indicated treatments of INS 832/13 cells as measured by qRT-PCR and presented as fold change relative to CTRL. (**c**) PFKFB3 immunostaining of INS 832/13 cells transduced with LacZ (CTRL) or hIAPP adenoviruses. (**d**) Quantification of PFKFB3 immunopositive cells in different treatments, shown as % of all cells. Data are presented as mean ± SEM, n=3 for each group. Statistical significance in b was analyzed by one-way ANOVA test with Tukey's post-test (***p<0.001, ****p<0.0001).

Supplementary Figure 12

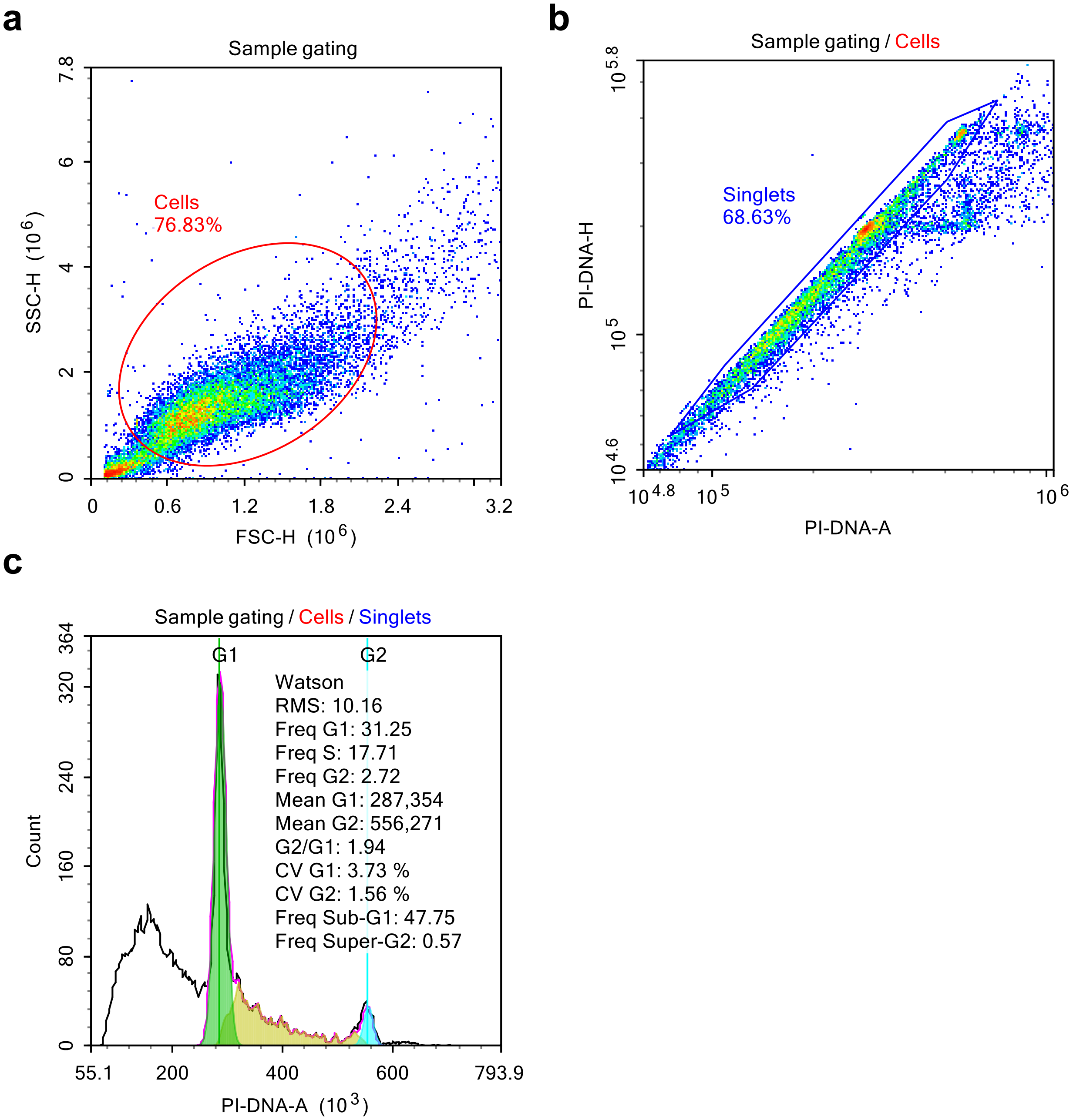

Supplementary Figure 12. Gating strategy used for cell cycle analysis of the DNA distribution. (a-c) Gating strategy to analyze cell cycle based on the DNA distribution involved singlet cell population that was analyzed by Watson algorithm in Supplementary Figure 3c and Supplementary Figure 10b.

SUPPLEMENTARY TABLE 1. Characteristics of WT and HIP rats used for islet isolation

ID	Genotype	Age (months)	Fasting blood glucose (mg/dl)
366-5	WT	2	54
366-7	WT	2	58
367-15	HIP	2	67
367-3	HIP	2	61
357-3	WT	4	64
358-1	WT	4	65
357-7	HIP	4	78
359-3	HIP	4	70
346-5	WT	6	62
257-7	WT	6	77
443-4	WT	6	82
442-6	WT	6	79
346-7	HIP	6	73
259-3	HIP	6	84
442-1	HIP	6	90
443-2	HIP	6	72
443-3	HIP	6	79

SUPPLEMENTARY TABLE 2. Characteristics of nPOD cases

	nPOD deidentified ID	Age (years)	Sex	BMI (kg/m^2)	Duration (years)	Treatment
Non-diabetic						
	6104	40-50	Male	20-40	–	–
	6288	50-60	Male	20-40	–	–
	6020	50-60	Male	20-40	–	–
T2D						
	6186	60-70	Male	20-40	5	Sitagliptin, Metformin
	6275	40-50	Male	20-40	2	None
	6255	50-60	Male	20-40	6	Metformin, Glibenclamide

SUPPLEMENTARY TABLE 3. Characteristics of ND and T2D islet donors from IIDP

	Received islets	Age (years)	Sex	BMI (kg/m^2)	Duration (years)	Treatment
Non-diabetic						
	1	40-50	Male	20-35	–	–
	2	20-30	Female	20-35	–	–
	3	30-40	Male	20-35	–	–
T2D						
	4	50-60	Male	20-35	8	None
	5	40-50	Male	20-35	>10	Diet, oral medications
	6	50-60	Female	20-35	0-5	Diet, oral medications
