# Supplementary material for: IAPP toxicity activates HIF1α/PFKFB3 signaling delaying β-cell loss at the expense of β-cell function
Source: Nat Commun. 2019 Jun 18;10:2679. doi: 10.1038/s41467-019-10444-1 (PMC6581914; doi:10.1038/s41467-019-10444-1)
Supplement: Supplementary file 3 — Reporting Summary [file 41467_2019_10444_MOESM3_ESM.pdf]

nature research

Corresponding author(s): DBPR NCOMMS-17-12269C

Last updated by author(s): Apr 26, 2019

Reporting Summary

Nature Research wishes to improve the reproducibility of the work that we publish. This form provides structure for consistency and transparency in reporting. For further information on Nature Research policies, see Authors & Referees and the Editorial Policy Checklist.

Statistics

For all statistical analyses, confirm that the following items are present in the figure legend, table legend, main text, or Methods section.

n/a	Confirmed	
☐	☒	The exact sample size (n) for each experimental group/condition, given as a discrete number and unit of measurement
☒	☐	A statement on whether measurements were taken from distinct samples or whether the same sample was measured repeatedly
☐	☒	The statistical test(s) used AND whether they are one- or two-sided<br>*Only common tests should be described solely by name; describe more complex techniques in the Methods section.*
☒	☐	A description of all covariates tested
☒	☐	A description of any assumptions or corrections, such as tests of normality and adjustment for multiple comparisons
☐	☒	A full description of the statistical parameters including central tendency (e.g. means) or other basic estimates (e.g. regression coefficient) AND variation (e.g. standard deviation) or associated estimates of uncertainty (e.g. confidence intervals)
☒	☐	For null hypothesis testing, the test statistic (e.g. F, t, r) with confidence intervals, effect sizes, degrees of freedom and P value noted<br>*Give P values as exact values whenever suitable.*
☒	☐	For Bayesian analysis, information on the choice of priors and Markov chain Monte Carlo settings
☒	☐	For hierarchical and complex designs, identification of the appropriate level for tests and full reporting of outcomes
☒	☐	Estimates of effect sizes (e.g. Cohen's d, Pearson's r), indicating how they were calculated

Our web collection on statistics for biologists contains articles on many of the points above.

Software and code

Policy information about availability of computer code

Data collection	N/A
Data analysis	N/A

For manuscripts utilizing custom algorithms or software that are central to the research but not yet described in published literature, software must be made available to editors/reviewers. We strongly encourage code deposition in a community repository (e.g. GitHub). See the Nature Research guidelines for submitting code & software for further information.

Data

Policy information about availability of data

All manuscripts must include a data availability statement. This statement should provide the following information, where applicable:

- Accession codes, unique identifiers, or web links for publicly available datasets
- A list of figures that have associated raw data
- A description of any restrictions on data availability

The data that support the findings of this study are available from the corresponding author upon reasonable request.

Field-specific reporting

Please select the one below that is the best fit for your research. If you are not sure, read the appropriate sections before making your selection.

☒ Life sciences ☐ Behavioural & social sciences ☐ Ecological, evolutionary & environmental sciences

For a reference copy of the document with all sections, see nature.com/documents/nr-reporting-summary-flat.pdf

Life sciences study design

All studies must disclose on these points even when the disclosure is negative.

Sample size	N/A
Data exclusions	No data exclusion
Replication	All reported data was replicated successfully
Randomization	N/A
Blinding	N/A

Reporting for specific materials, systems and methods

We require information from authors about some types of materials, experimental systems and methods used in many studies. Here, indicate whether each material, system or method listed is relevant to your study. If you are not sure if a list item applies to your research, read the appropriate section before selecting a response.

Materials & experimental systems

n/a	Involved in the study	
☐	☒	Antibodies
☐	☒	Eukaryotic cell lines
☒	☐	Palaeontology
☐	☒	Animals and other organisms
☒	☐	Human research participants
☒	☐	Clinical data

Methods

n/a	Involved in the study	
☒	☐	ChIP-seq
☐	☒	Flow cytometry
☒	☐	MRI-based neuroimaging

Antibodies

Antibodies used

For detection of PFKFB3, we utilized a previously reported antibody75, anti-PFKFB3 (Abcam 181861, Cambridge, UK, 1:200 for IF, 1:1000 for WB) the specificity of which we confirmed by silencing PFKFB3 and measuring transcript and protein levels by western blot, qRT-PCR, and immunofluorescence (Supplementary Figure 11A-D).

The following antibodies were used: anti-PFKFB3 (Abcam 181861, Cambridge, UK,1:400 for IHC, 1:200 for IF, 1:1000 for WB), anti-Tom20 (Santa Cruz Biotechnology sc-11415, Dallas, TX, USA, 1:200 for IF), anti-HIF1α (NOVUS Biologicals NB100-105, Littleton, CO, USA, 1:1000 for WB), anti-MFN2 (Cell Signaling Technology 9482S, Danvers, MA, USA, 1:1000 for WB), anti-Opa1 (BD Transduction 612606, San Diego, CA, USA, 1:1000 for WB), anti-Drp1 (Cell Signaling Technology 8570S, Danvers, MA, USA, 1:1000 for WB), anti-nucleolin (Santa Cruz Biotechnology sc-13057, Dallas, TX, USA, 1:1000 for WB), anti-PARP1 (Cell Signaling Technology 9542S, Danvers, MA, USA, 1:1000 for WB), anti-insulin (DAKO A0564, Glostrup, Denmark, 1:200 for IF), anti-GAPDH and anti-cleaved caspase 3 from Cell Signaling Technology 2118S and 9664S, respectively, Danvers, MA, USA, (1:1000 for WB).
Secondary antibodies for immunofluorescence staining were F(ab')2 conjugates with Cy3 or FITC purchased from Jackson Laboratories and used at dilution of 1:200.
Uncropped and unprocessed Western blots are provided by a separate Source Data file.

Validation

For detection of PFKFB3, we utilized a previously reported antibody75, anti-PFKFB3 (Abcam 181861, Cambridge, UK, 1:200 for IF, 1:1000 for WB) the specificity of which we confirmed by silencing PFKFB3 and measuring transcript and protein levels by western blot, qRT-PCR, and immunofluorescence (Supplementary Figure 11A-D).
All the other antibodies are commercially purchased and validation is provided at their specific website by the vendor.

Eukaryotic cell lines

Policy information about cell lines

Cell line source(s)	INS 832/13 rat beta cell line is a gift from Dr. Chris Newgard from Duke University
Authentication	Not authenticated
Mycoplasma contamination	Not tested
Commonly misidentified lines (See ICLAC register)	Not such cell line used

Animals and other organisms

Policy information about studies involving animals; ARRIVE guidelines recommended for reporting animal research

Laboratory animals	Male rats transgenic for hIAPP (HIP) and WT counterparts were used at 2-6 months age
Wild animals	N/A
Field-collected samples	N/A
Ethics oversight	Animal Research Commitee at UCLA approved the breeding and experimental protocols

Note that full information on the approval of the study protocol must also be provided in the manuscript.

Flow Cytometry

Plots

Confirm that:

- [ ] The axis labels state the marker and fluorochrome used (e.g. CD4-FITC).
- [x] The axis scales are clearly visible. Include numbers along axes only for bottom left plot of group (a 'group' is an analysis of identical markers).
- [ ] All plots are contour plots with outliers or pseudocolor plots.
- [x] A numerical value for number of cells or percentage (with statistics) is provided.

Methodology

Sample preparation	INS 832/13 cells after various treatments were collected with trypsinization, washed with ice-cold PBS, pelleted at 900 g and then fixed with 80% methanol for 2h at -20C. Cells were pelleted, washed with ice-cold PBS, again pelleted and then resuspended in a solution containing 50 ug/ml propidium iodide and 50 ug/ml RNAse A. Cell suspensions were incubated for 1 hour at 37C before FACS analysis of the DNA distribution.
Instrument	NovoCyte 1000 (ACEA Biosciences)
Software	NovoExpress (ACEA Biosciences)
Cell population abundance	N/A
Gating strategy	Cell cycle analysis was performed on the singlet cell population identified on scatters plot. The analysis of the cell cycle distribution was based on the Watson algorithm.

- [x] Tick this box to confirm that a figure exemplifying the gating strategy is provided in the Supplementary Information.
