# Supplementary material for: IAPP toxicity activates HIF1α/PFKFB3 signaling delaying β-cell loss at the expense of β-cell function
Source: Nat Commun. 2019 Jun 18;10:2679. doi: 10.1038/s41467-019-10444-1 (PMC6581914; doi:10.1038/s41467-019-10444-1)
Supplement: Supplementary file 4 — Source Data [file 41467_2019_10444_MOESM4_ESM.zip › Source Data.pptx]

## Slide 1
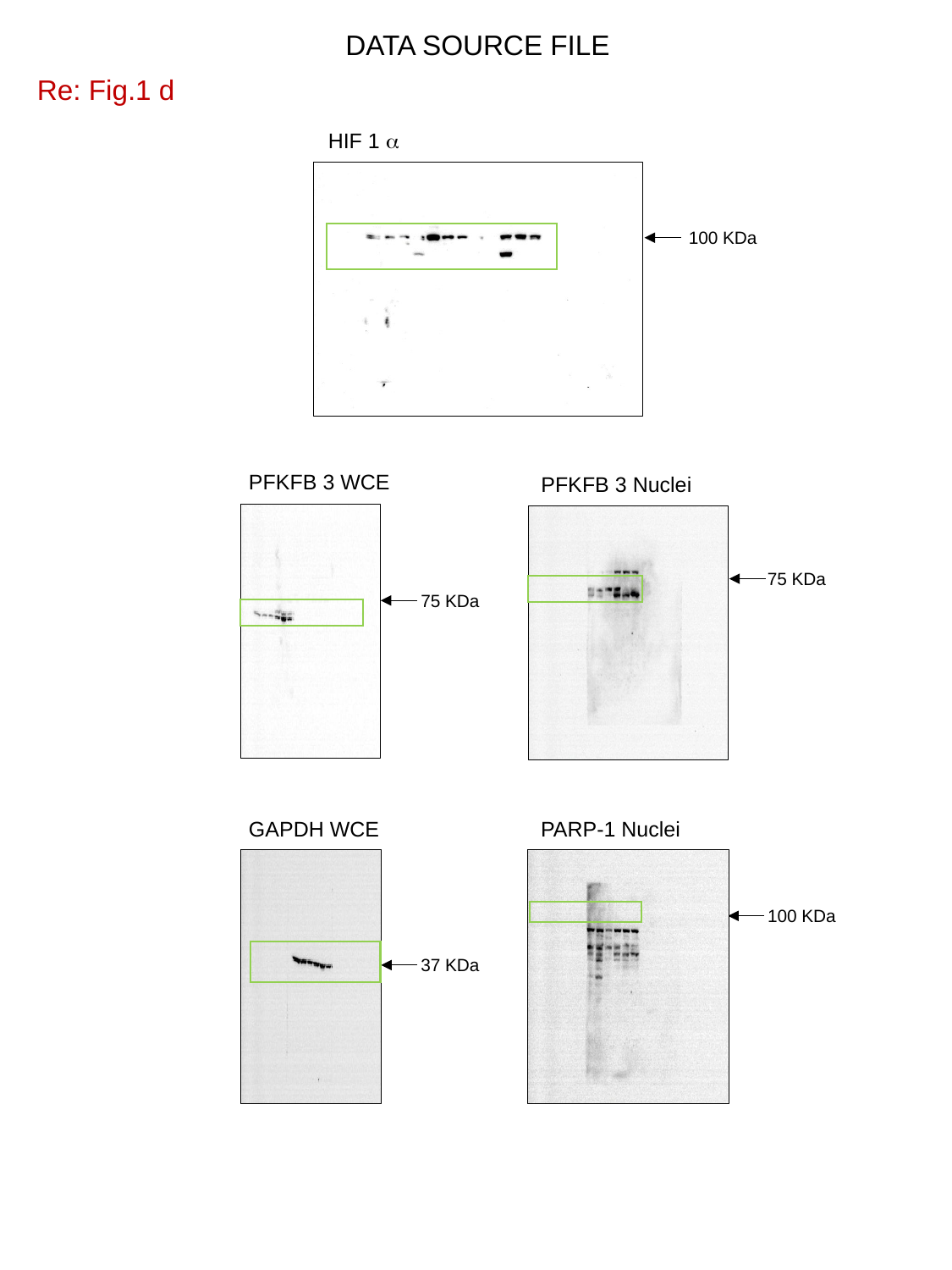

DATA SOURCE FILE
Re: Fig.1 d
HIF 1 a
100 KDa
PFKFB 3 WCE
PFKFB 3 Nuclei
75 KDa
75 KDa
GAPDH WCE
PARP-1 Nuclei
100 KDa
37 KDa

## Slide 2
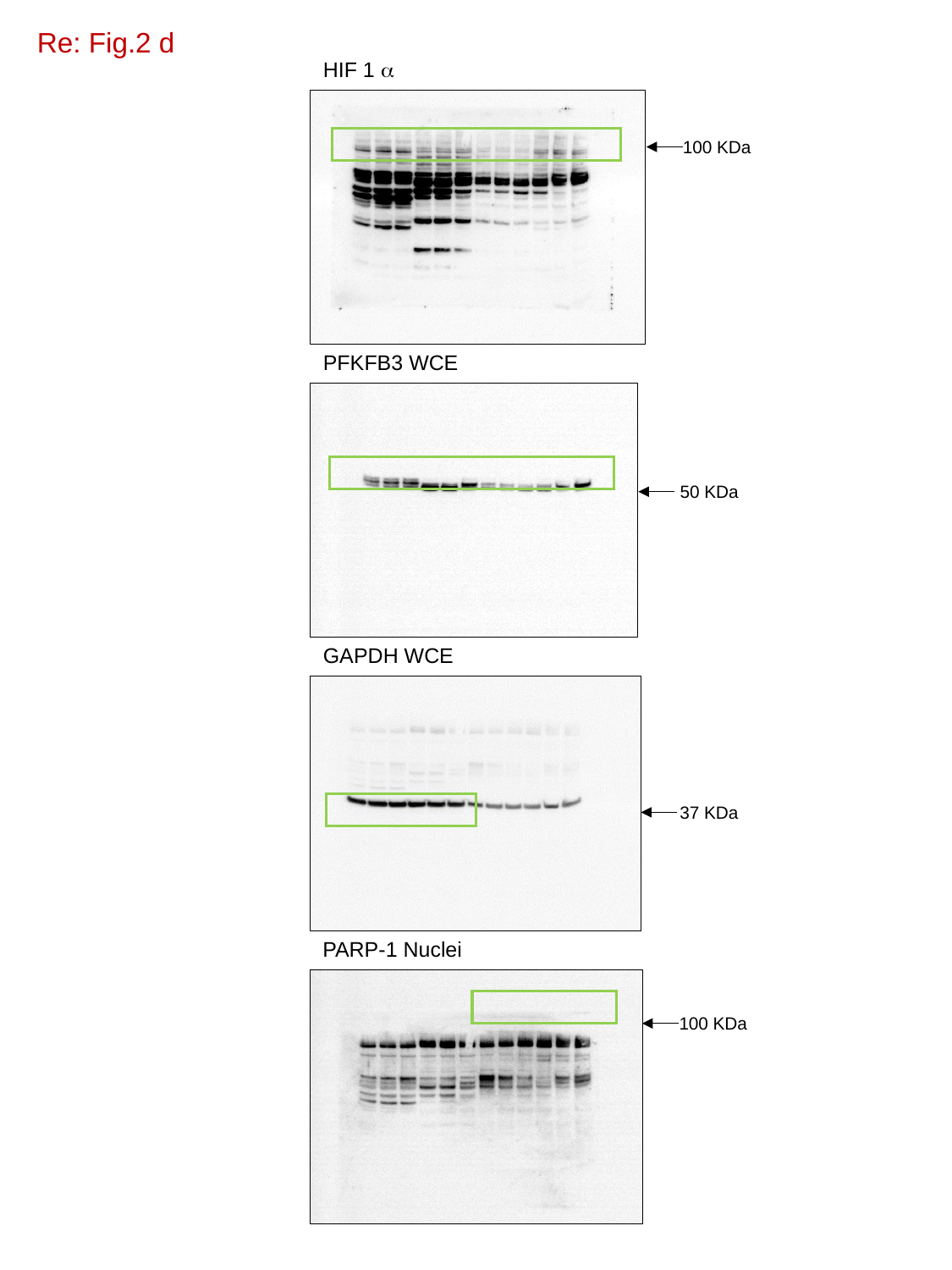

Re: Fig.2 d
HIF 1 a
100 KDa
PFKFB3 WCE
50 KDa
GAPDH WCE
37 KDa
PARP-1 Nuclei
100 KDa

## Slide 3
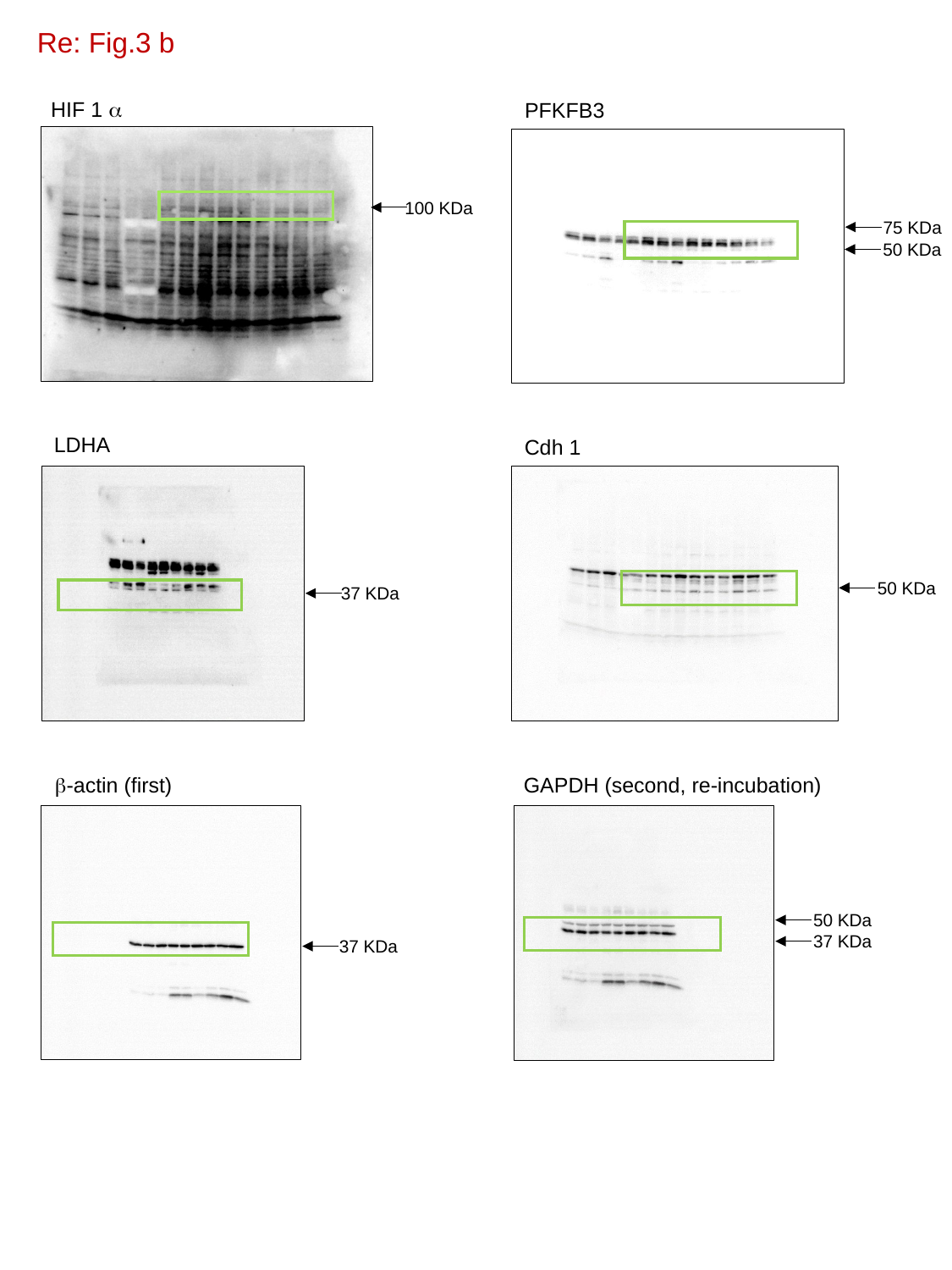

Re: Fig.3 b
HIF 1 a
PFKFB3
100 KDa
75 KDa
50 KDa
LDHA
Cdh 1
50 KDa
37 KDa
GAPDH (second, re-incubation)
50 KDa
37 KDa
b-actin (first)
37 KDa

## Slide 4
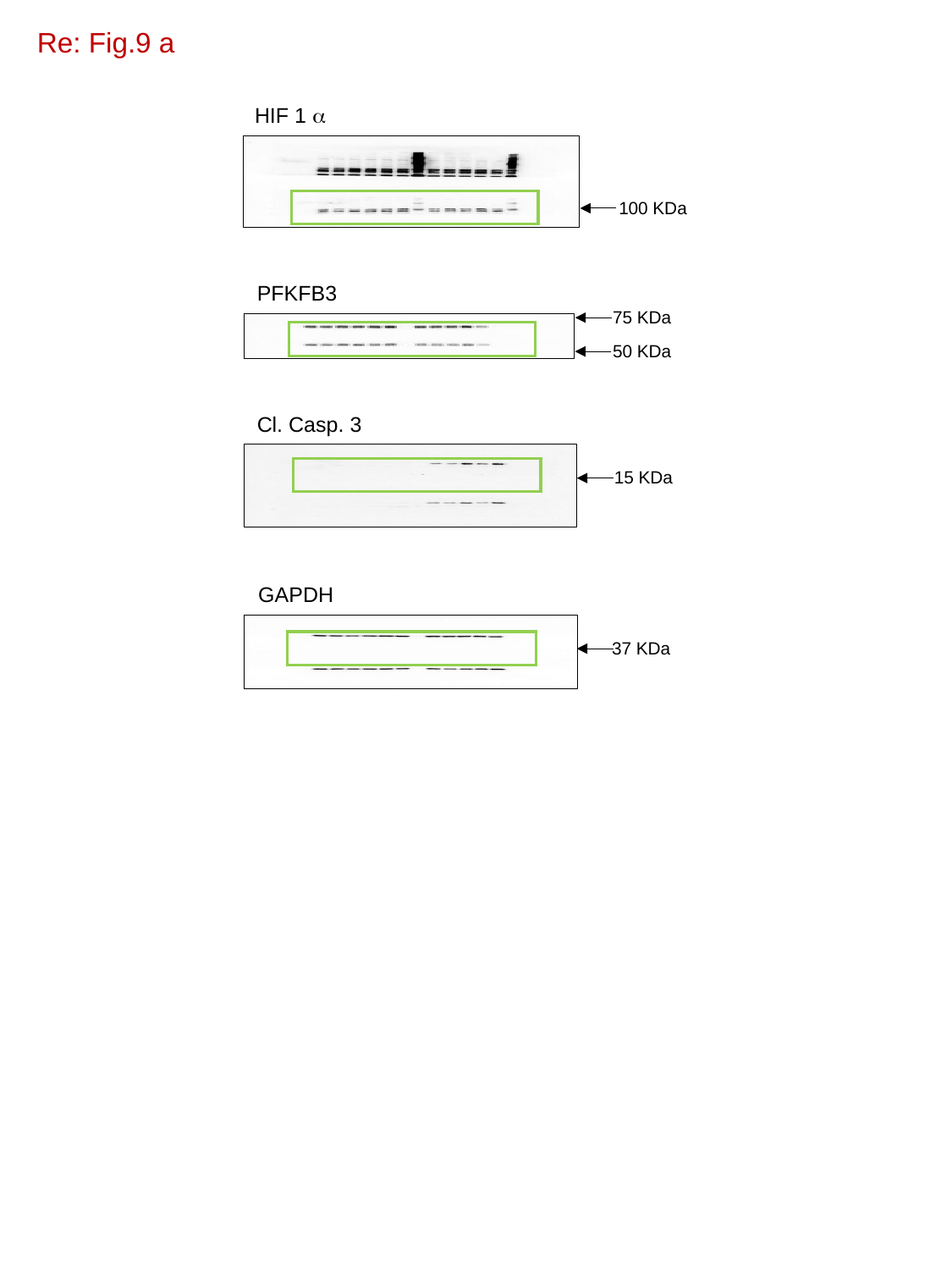

Re: Fig.9 a
HIF 1 a
100 KDa
PFKFB3
75 KDa
50 KDa
Cl. Casp. 3
15 KDa
GAPDH
37 KDa
